# Development of a potency assay for CD34^+^ cell-based therapy

**DOI:** 10.1038/s41598-023-47079-8

**Published:** 2023-11-11

**Authors:** Anne Aries, Christine Vignon, Céline Zanetti, Aurélien Goubaud, Arthur Cormier, Anne Diederichs, Rachid Lahlil, Philippe Hénon, Ibon Garitaonandia

**Affiliations:** 1 Institut de Recherche en Hématologie et Transplantation, Hôpital du Hasenrain, 87 Avenue d’Altkirch, Mulhouse, France; 2 CellProthera SAS, 12 Rue du Parc, Mulhouse, France

**Keywords:** Haematopoietic stem cells, Cardiology

## Abstract

We have previously shown that intracardiac delivery of autologous CD34^+^ cells after acute myocardial infarction (AMI) is safe and leads to long term improvement. We are now conducting a multicenter, randomized, controlled Phase I/IIb study in post-AMI to investigate the safety and efficacy of intramyocardial injection of expanded autologous CD34^+^ cells (ProtheraCytes) (NCT02669810). Here, we conducted a series of in vitro studies characterizing the growth factor secretion, exosome secretion, gene expression, cell surface markers, differentiation potential, and angiogenic potential of ProtheraCytes clinical batches to develop a potency assay. We show that ProtheraCytes secrete vascular endothelial growth factor (VEGF) and its concentration is significantly correlated with the number of CD34^+^ cells obtained after expansion. ProtheraCytes also secrete exosomes containing proangiogenic miRNAs (126, 130a, 378, 26a), antiapoptotic miRNAs (21 and 146a), antifibrotic miRNAs (133a, 24, 29b, 132), and miRNAs promoting myocardial regeneration (199a and 590). We also show that ProtheraCytes have in vitro angiogenic activity, express surface markers of endothelial progenitor cells, and can differentiate in vitro into endothelial cells. After the in vitro characterization of multiple ProtheraCytes clinical batches, we established that measuring the concentration of VEGF provided the most practical, reliable, and consistent potency assay.

## Introduction

Acute myocardial infarction (AMI) inflicts massive damage leading to coronary artery occlusion and progressive cell death in the infarct region^1^. AMI affects around 126 million individuals globally and wound repair after AMI involves a robust angiogenesis response to promote vessel formation and tissue repair^2^. Ample clinical evidence collected for more than two decades, indicates that cell therapies can induce revascularization and regeneration of infarct tissue after heart failure^3,4^. Stem cells^5,6^, extracellular vesicles^7^ and secretome-based therapies^8^ have been shown to improve angiogenesis and tissue repair after MI. The beneficial effect of cell transplantation is likely due, at least in part, by the secretion of paracrine factors that may promote cardiomyocyte survival and angiogenesis^5^. But still, these therapies have not demonstrated definitive efficacy in myocardial infarction and heart failure and additional preclinical and clinical studies are necessary.

Human CD34^+^ stem cells, obtained from blood or bone marrow have already shown promising results in cardiac regenerative medicine, in addition to their effectiveness in the treatment of blood diseases. Indeed, intramyocardial injection of autologous peripheral blood CD34^+^ stem cells into the damaged myocardium of patients with very low prognosis, allowed a significant long-term restoration of cardiac function^3^. The improvement was such that three patients initially recommended for early heart transplantation, no longer required it years after the CD34^+^ cell injection. Moreover, these cells have been shown to secrete factors that promote angiogenesis and therefore preserve the function of the ischemic myocardium^9^. In other ischemic indications such as critical limb ischemia, transplantation of autologous CD34^+^ cells led to improvements in Wong-Baker FACES pain rating scale, transcutaneous partial oxygen pressure, total or pain-free walking distance, and ulcer size^10^.

The postulated mechanism of action by which CD34^+^ cells trigger an angiogenic response is through their paracrine activity. CD34^+^ cells secrete regulators of vasculogenesis including growth factors such as vascular endothelial growth factor (VEGF) and exosomes containing pro-angiogenic miRNAs. VEGF is expressed in multiple tissues and cells, including CD34^+^ stem cells^11^ and is a potent mediator of both angiogenesis and vasculogenesis^12,13^. VEGF is required for regeneration of vascular injury in damaged tissues and plays a crucial role in the recruitment of endogenous endothelial cells which subsequently undergo proliferation and differentiation into new blood vessels^14^. Secretion of pro-angiogenic vascular growth factors and cytokines such as VEGF, SDF-1, Angiopoietin 1, PlGF, FGF-2, NGF and IL-1β influences favorably the angiogenic function of endothelial progenitor cells, increases chemotactic cell migration, and reduces apoptosis^11,15^.

There is growing evidence suggesting that miRNAs have proangiogenic activities^16^ and exosomes from CD34^+^ cells have been shown to be enriched in proangiogenic microRNA 126 and 130a in comparison to CD34^+^ cells^17^. Other miRNAs including miR-199a, miR-302b, miR-518, miR-590 and miR-1825 have been involved in the regulation of cardiac reprogramming and regeneration^18^.

The development of advanced therapeutic medicinal products (ATMPs) for cell transplantation requires a potency assay before commencing the pivotal studies and commercialization of the ATMP. This potency assay should quantitatively measure the relevant biological product attribute, have lot to lot consistency, and be based on the ATMP’s mechanism of action for the particular disease intended to treat. In order to develop a potency assay, we conducted a series of in vitro studies characterizing the growth factor secretion, exosome secretion, gene expression, cell surface markers, differentiation potential, and angiogenic potential of ProtheraCytes clinical batches, which are human autologous CD34^+^ cells expanded according to a GMP automated manufacturing process designed for large clinical scale production^19,20^. The potency assay is a critical quality control measure required to determine whether the expanded cells possess the characteristics that will promote cardiac regeneration and revascularization and should be an easy and simple method that allows for the quick release of the batch before injection in the clinic.

## Methods

### Cell culture

ProtheraCytes were obtained after expansion of mobilized CD34^+^ cells from AMI patients (EXCELLENT Phase I/IIb clinical trial NCT02669810) and from Frozen Healthy Donors (FHD) CD34^+^ purified cells (Lonza, NC, USA) as previously described^19^. Informed consent was obtained from all subjects involved in the study. The study was conducted according to the guidelines of the Declaration of Helsinki.

### VEGF quantification

Supernatants from AMI patients and FHD ProtheraCytes were collected after 9 days of expansion in StemFeed cell culture medium (Eurobio, France) and stored at −80 °C until analysis. Supernatants were thawed and VEGF levels were measured using the Human VEGF QuantiGlo ELISA Kit (R&D Systems, MN, USA) according to the manufacturer’s instructions with the SpectraMax L (Molecular Devices, San Jose, CA USA). The Immunoassay Control Set 732 for Human VEGF (R&D Systems) was used as a positive control and StemFeed medium as a negative control.

### In vitro tube formation assay

2.5 × 10^4^ serum-starved overnight Human umbilical Vein Endothelial Cells (HUVECs) were seeded with ProtheraCytes supernatant or StemFeed medium as negative control into 48-well plates that had been coated with 150 µL of growth-factor–reduced Matrigel (Corning, Arizona, US). And to confirm that VEGF was responsible for the angiogenic activity, 2.5 × 10^4^ serum-starved overnight HUVECs were also cultured with 1 ng/mL VEGF (concentration in the range of the ProtheraCytes samples) in PBS buffer or PBS buffer as negative control into 48-well plates that had been coated with 150 µL of growth-factor–reduced Matrigel.

Tube formation was examined by phase-contrast microscopy 6 h later and the number of tubes was quantified in each condition in triplicate.

### Exosome isolation from ProtheraCytes

ProtheraCytes were seeded in a serum-free medium (DMEM/F12 medium without phenol red, Thermo Fisher) in a bioreactor for extracellular vesicle release by exerting a controlled mechanical stimulation on cells for 30 min to 2 h according to Everzom’s proprietary method^21^. The size distribution and concentration measurements of ProtheraCytes-derived exosomes were performed using the Nanosight (NS300, Malvern, UK). The exosome membrane markers were quantified by flow cytometry using the MACSPlex Exosome kit (Miltenyi).

For miRNA analysis, ProtheraCytes were cultured at the concentration of 2.5 × 10^5^ cells/mL in StemSpan-AOF (StemCell Technologies, BC Canada) supplemented with the same cytokines used for the ProtheraCytes culture^19^ for 40 h. Then, cells were collected by centrifugation at 400*g* for 10 min; and exosomes were purified from the supernatant by precipitation using the ExoQuick-TC kit (System Biosciences, CA, USA) according to the manufacturer’s instructions. After isolation, exosomes were characterized by flow cytometry using the ExoStep kit with a bead-bound anti-CD63 capture, anti-CD81 and anti-CD34 antibodies (ImmunoStep, Spain) confirming the identity of the exosomes secreted by ProtheraCytes.

### MicroRNA quantification

Total RNA from ProtheraCytes and their secreted exosomes collected from 7 AMI patients were isolated respectively using a miRNeasy Tissue/cells Advanced MiniKit and a miRNeasy Serum/Plasma Advanced Kit (QiAGEN, France) according to the manufacturer’s protocols. RNA isolated from exosomes and cells was reverse transcribed to cDNA using the miCURY LNA RT Kit (QIAGEN, France). UniSp6 RNA spike-in controls were added during cDNA synthesis to ensure the quality of the experiment. Real-time qPCR amplifications were performed for each RT reaction. Reactions were performed according to the manufacturers’ instructions using a miRCURY LNA miRNA SYBR Green PCR Kit (QIAGEN, France) with the Bio-Rad CFX96 Real time PCR Detection System (BioRad Laboratories, France). All primer sets were custom designed by the supplier. Primers used were miR-21-5p (YP00204230), miR-26a-5p (YP00206023), miR-126-3p (YP00204227), miR-130a-3p (YP002046658), miR-133a-3p (YP00204788), miR-146a-5p (YP00204688), miR-199a-3p (YP00204536), miR-378a-3p (YP00205946), miR-590-3p (YP00205448), miR-24-3p (YP00204260), miR-29b-3p (YP00204679) and miR-132-3p (YP00206035), miR-34a-3p (YP00206061), miR-143-3p miRNA (YP00205992), and miR-506-3p (YP00204539). The qPCR data were normalized to miR-let7a-5p (YP00205727) values. Relative miRNA expressions were calculated using the 2^-ΔΔCt^ method.

### ProtheraCytes transcriptomic analysis

Total RNA was purified from ProtheraCytes collected from 7 FHD and 8 AMI patients using RNAeasy plus minikit (Qiagen, Courtabeuf, France). Briefly, sample quality was assessed using a DNA High sensitivity chip (Agilent Technologies). Single-end 50 reads barcoded RNA-Seq sequencing library was performed on an Illumina HiSeq4000 with the TruSeq SBS v3 chemistry at iGE3 Genomics Platform (University of Geneva). The normalization and differential expression analysis was performed with the R/Bioconductor package edgeR v.3.28.1, for the genes annotated in the reference genome. Lowly expressed genes were filtered, keeping genes that are expressed at a reasonable level (10 counts in at least 7 samples). The filtered data were normalized by the library size. The differentially expressed genes were estimated with the GLM approach (Generalized Linear Model) using a negative binomial distribution. The genes were considered as differentially expressed when the fold change (FC) was at least twofold with a 5% false discovery rate (FDR) Benjamini–Hochberg multiple testing correction. The raw data was uploaded to GEO (accession# GSE220865).

### Flow cytometry analysis

ProtheraCytes from AMI patients were harvested, and then washed once with cold PBS, and after centrifugation, cell pellets were suspended in PBS. The number of cells in each sample was adjusted to 1–2 × 10^5^ cells/100 µL. Cells were incubated for 20 min at 4 °C in the dark with pre-conjugated antibodies for flow cytometry analysis of surface markers commonly found in stem cells and early endothelial progenitors. Conjugated antibodies used included FITC anti-CD34 (clone AC136), APC anti-CD31 (REA730), PEVio770 anti-CD133 (REA753), PE anti-CD184 (REA649), PEVio770 anti-CD117 (REA787), PE anti-CD49b (REA188) (Miltenyi Biotec). Isotype controls were used to validate the specificity of antibodies. For dead cell discrimination in flow cytometry analysis, cells were incubated with 7-AAD (Miltenyi Biotec). The flow cytometry analysis was performed using a BD FACSLyric (Becton Dickinson Biosciences).

### Endothelial differentiation of ProtheraCytes

ProtheraCytes from frozen healthy donors were seeded on fibronectin coated plates at a density of 1 × 10^5^ cells/cm^2^ and cultured in endothelial cell growth medium-2 EGM2 (Lonza) supplemented with 20% FBS (Eurobio) in the presence of 50 nM of UM171 and 2.5 µM nicotinamide acid (NMA) (all from StemCell Technologies, France) with the addition of 5 ng/ml bone morphogenic protein 4 (BMP4) and 10 ng/ml human basic fibroblast growth factor 2 (FGF2) (Miltenyi Biotec, France). Three days later, cells were differentiated to the endothelial lineage as described previously^22^. Briefly, these cells were first differentiated by adding 10 µM CHIR99021 (StemCell Technologies, France), a GSK3 inhibitor, to the medium for 3 days. Endothelial specification was induced on the following day by culturing the cells in EGM2 medium supplemented with 35 nM UM171, 2.5 µM Nicotinamide acid (NMA), 300 ng/mL VEGF (Myltenyi Biotec, France), and 2 µM forskolin (StemCell Technologies, France). The medium was changed every two days thereafter, until the confluency of the culture reached 90–100%. After the first passage, endothelial cell commitment and differentiation were maintained with 1 µM SB431542 (StemCell Technologies, France), a TGFβ inhibitor, and medium was changed every 2–3 days until cell analysis after 20–25 days of culture.

### Immunofluorescence labelling of ProtheraCytes induced to endothelial differentiation

ProtheraCytes immobilized on slides after cytospin centrifugation and ProtheraCytes undergoing endothelial differentiation in chamber slides were fixed in methanol for 10 min at 4 °C and blocked in 5% BSA for 1 h at room temperature before antibodies were added. Primary antibodies were specific for CD31 (Ozyme), von Willebrand factor (vWF, Agilent Technologies, France), Vascular Endothelial Growth Factor Receptor 2 (VEGFR-2, Miltenyi Biotech France), and Vascular cell adhesion protein 1 (VCAM-1, Abcam, Netherlands). Secondary antibodies used were goat anti-Rabbit Alexa Fluor 488 (Fischer Scientific, France), goat anti-mouse Alexa Fluor 488 (Fisher Scientific, France) and goat anti-mouse Alexa Fluor 555 (Fisher Scientific, France). DAPI (CliniSciences, France) was used to visualize nuclei. Staining was analyzed by fluorescence microscopy.

### Statistical analysis

GraphPad Prism and Excel were used to generate the graphs. All quantitative data-points represent the mean ± standard error of the mean (SEM). Statistical significance was identified through Analysis of variance (ANOVA) and Student’s t-test or Mann–Whitney test were used to compare mean variables of two cohorts.

### Ethics approval and consent to participate

All human tissues were obtained with written informed consent. The clinical study titled: EXCELLENT (Expanded CELL Endocardiac Transplantation) A multicentric controlled phase I/IIb study evaluating the safety and the efficacy of in vitro expanded peripheral blood CD34^+^ stem cells output by the StemXpand Automated Process, and injected in patients with an acute myocardial infarction and a LVEF remaining below 50% versus standard of care (ClinicalTrials.gov Identifier: NCT02669810). The clinical study was approved by the Comité de Protection des Personnes du Sud-Ouest et Outre-Mer IV ethics committee on 11th of December 2015 (reference number# 14.00115.201428). The study was conducted according to the guidelines of the Declaration of Helsinki.

## Results

### Secretion of VEGF as a potency assay

**Figure 1:**
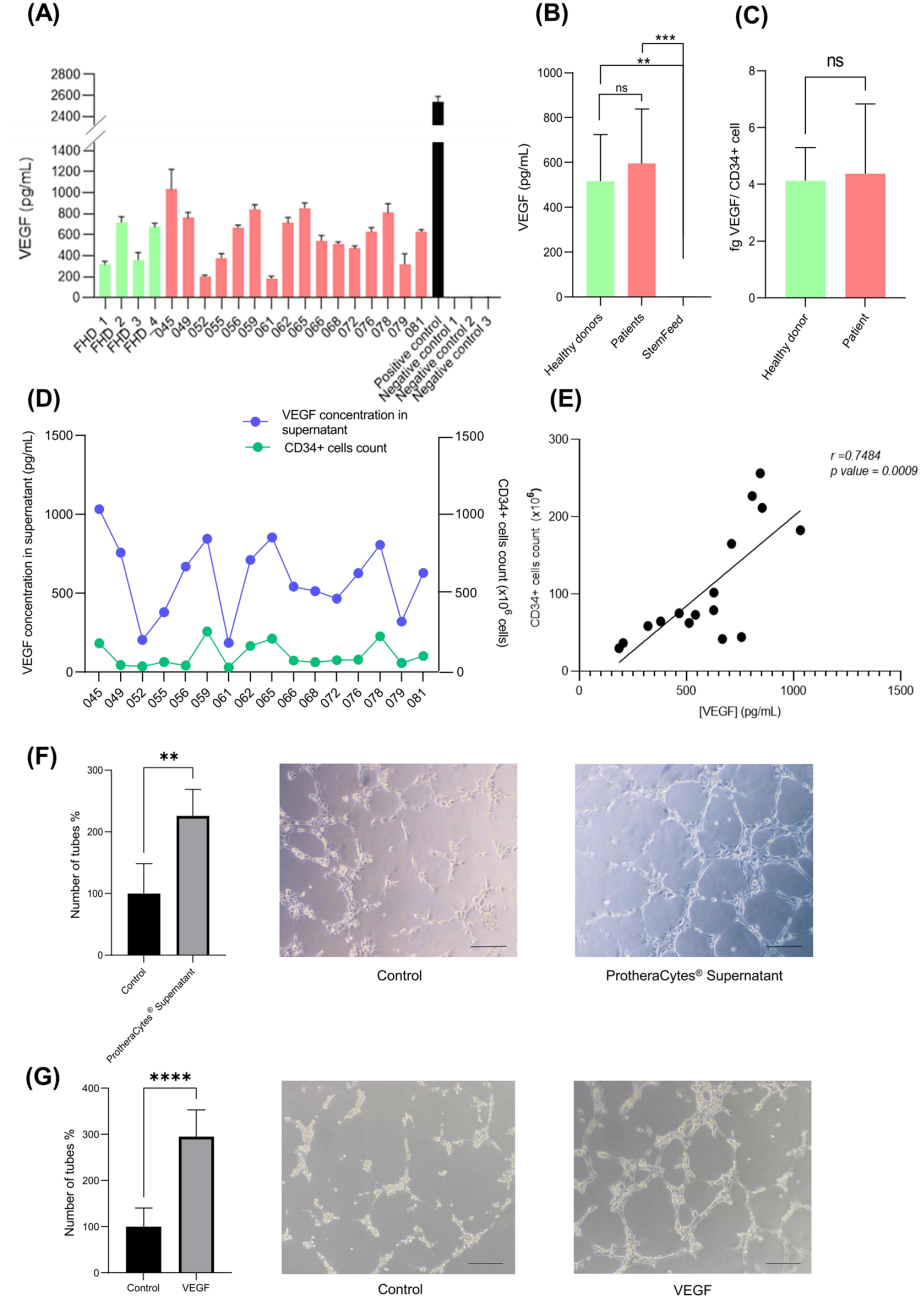
Quantification of VEGF in the supernatant of ProtheraCytes cultures as a potency assay. (**A**) Concentration of VEGF in cell culture supernatants after 9 days of CD34^+^ cell expansion from four healthy donors and 16 patients with acute myocardial infarction (EXCELLENT study). (**B**) No significant difference observed when VEGF concentration was compared between patients and healthy donors, but a significant difference was observed between patients and StemFeed (t-test, p = 0.0007) and healthy donors and StemFeed (t-test, p = 0.0087). (**C**) When the VEGF concentration was normalized by the number of CD34^+^ cells, we observed that there was no significant difference between the VEGF secreted per cell in healthy donors (4.1 fg/cell) and AMI patients (4.4 fg/cell) (p = 0.6343). (**D**) VEGF concentration and number of CD34^+^ cells after expansion. (**E**) Significant correlation between VEGF concentration and the number of CD34^+^ cells after expansion (Pearson correlation coefficient = 0.7484, p value = 0.0009). (**F,G**) In vitro tube formation assays: (**F**) HUVECs cocultured with ProtheraCytes supernatant have significantly higher tube formation than HUVECs cocultured with StemFeed control (t-test, p = 0.0082). (**G**) HUVECs cocultured with VEGF in PBS buffer have significantly higher tube formation than HUVECs cocultured with PBS control (t-test, p < 0.0001). The pictures display representative brightfield images of tube formation. Scale bar, 20 µm.

Human CD34^+^ cells have been shown to secrete VEGF^11^ and we wanted to determine the level of VEGF secretion by CD34^+^ cells after expansion of 16 ProtheraCytes batches manufactured from AMI patients from the EXCELLENT Phase I/IIb clinical trial, as well as 4 batches manufactured from healthy donors. The quantification of VEGF concentration by ELISA showed that the culture supernatants of ProtheraCytes from patients ranged from 185.6 to 1032.4 pg/mL with a mean value of 596.2 ± 242.3 pg/mL, and the VEGF concentration from healthy donor cells ranged from 315.3 to 718.3 pg/mL with a mean value of 526.2 ± 208.1 pg/mL (Fig. 1A). No significant difference was observed between the VEGF concentrations of patients and healthy donors (Fig. 1A, B). Conversely, the concentration of VEGF observed in the StemFeed culture medium (negative control) ranged from 2.7 to 3.0 pg/mL with a mean value of 2.8 ± 0.2 pg/mL, which was significantly lower than the VEGF concentration of patients (p = 0.0007) and healthy donors (p = 0.087) (Fig. 1B). When the VEGF concentration was normalized by the number of CD34^+^ cells, the VEGF secreted per cell in AMI patients was 4.4 fg/cell and was not significantly different (p = 0.6343) from the VEGF secreted per cell in healthy donors (4.1 fg/cell) (Fig. 1C). Furthermore, the concentration of VEGF in the culture supernatant of the expanded CD34^+^ cells from AMI patients was significantly correlated with the number of CD34^+^ cells obtained after expansion (Fig. 1D, E) (Pearson correlation coefficient r = 0.7484; p-value = 0.0009).

We then conducted an in vitro tube formation assay with HUVECs to determine if the ProtheraCytes supernatant containing VEGF has angiogenic activity. We quantified tube formation in HUVECs that had been cocultured for 6 h with ProtheraCytes supernatant or StemFeed medium as negative control and observed significantly higher tube formation in HUVECs cocultured with ProtheraCytes supernatant (p = 0.0082) (Fig. 1F). And to confirm that VEGF was responsible for the angiogenic activity observed with the ProtheraCytes samples, a tube formation assay was performed with 1 ng/mL VEGF in PBS buffer or PBS buffer as negative control. We also observed significantly higher tube formation in HUVECs cultured with 1 ng/mL VEGF (p < 0.0001), which is a concentration in the range of the ones obtained with the ProtheraCytes samples (Fig. 1G).

### ProtheraCytes-derived exosome characterization

**Figure 2:**
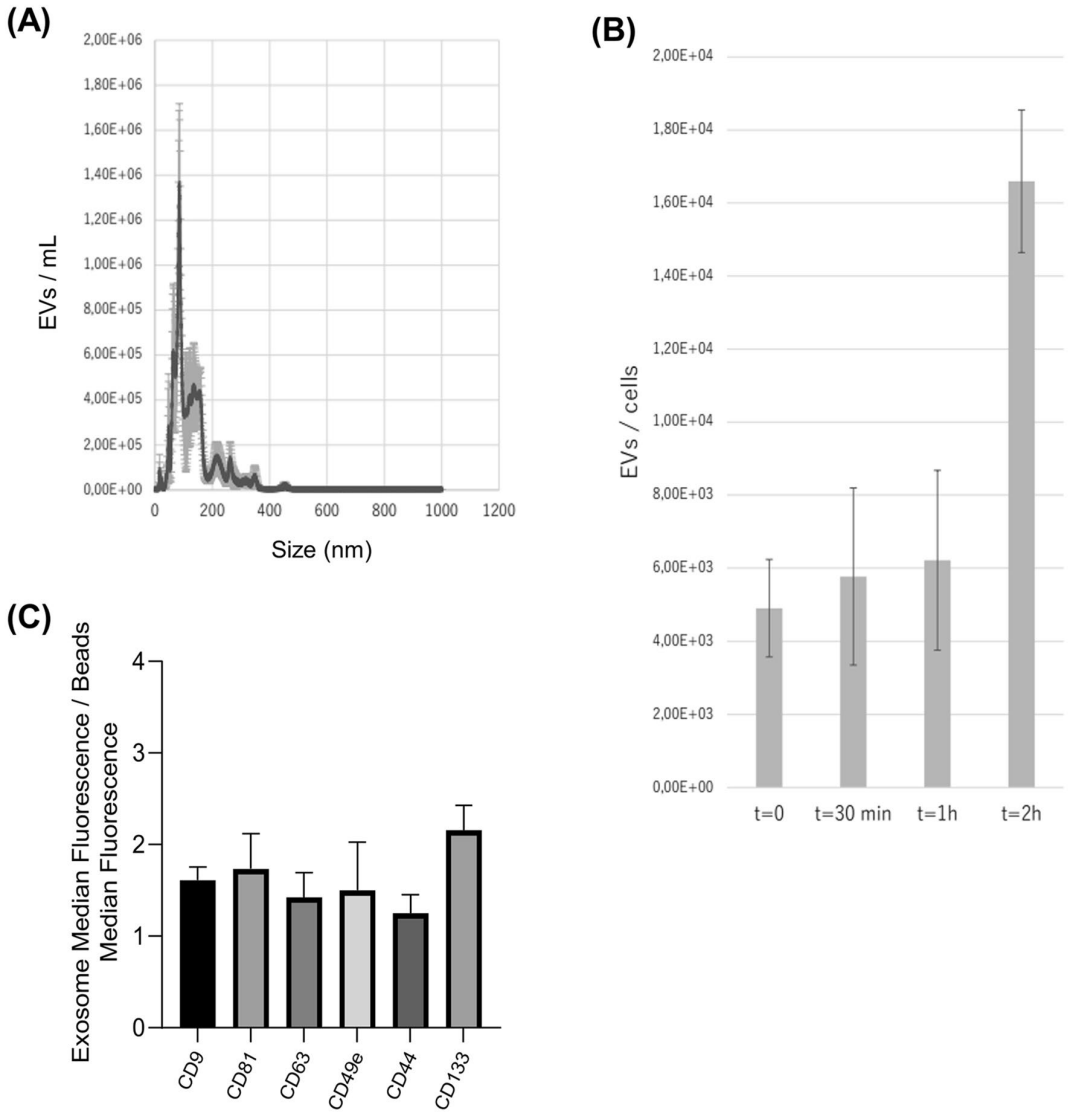
Characterization of ProtheraCytes-derived exosomes. (**A**) The average size of ProtheraCytes-derived exosomes was determined at 86.7 ± 10.17 nm. (**B**) Exosomes secreted by ProtheraCytes increased over time from around 6000 extracellular vesicles/cell at 30 min to 16,000 extracellular vesicles/cell at 2 h. (**C**) ProtheraCytes-derived exosomes express the characteristic exosomal markers CD63, CD81 and CD9 as well as endothelial (CD49b and CD44) and stem cell markers (CD133).

In collaboration with Everzom, ProtheraCytes were seeded in a bioreactor with controlled mechanical stimulation to collect exosomes. The average size of Protheracytes-derived exosomes was 86.7 ± 10.17 nm (Fig. 2A). We observed that the number of exosomes secreted by ProtheraCytes increased over time from around 6000 extracellular vesicles/cell at 30 min to 16,000 extracellular vesicles/cell at 2 h (Fig. 2B). We also analyzed the marker expression of the exosomes and showed that they express the exosomal markers CD9, CD63, and CD81 as well as the endothelial CD49b, CD44 endothelial, CD133 stem cell markers (Fig. 2C).

### MicroRNA expression in ProtheraCytes^-^derived exosomes (CD34Exo)

**Figure 3:**
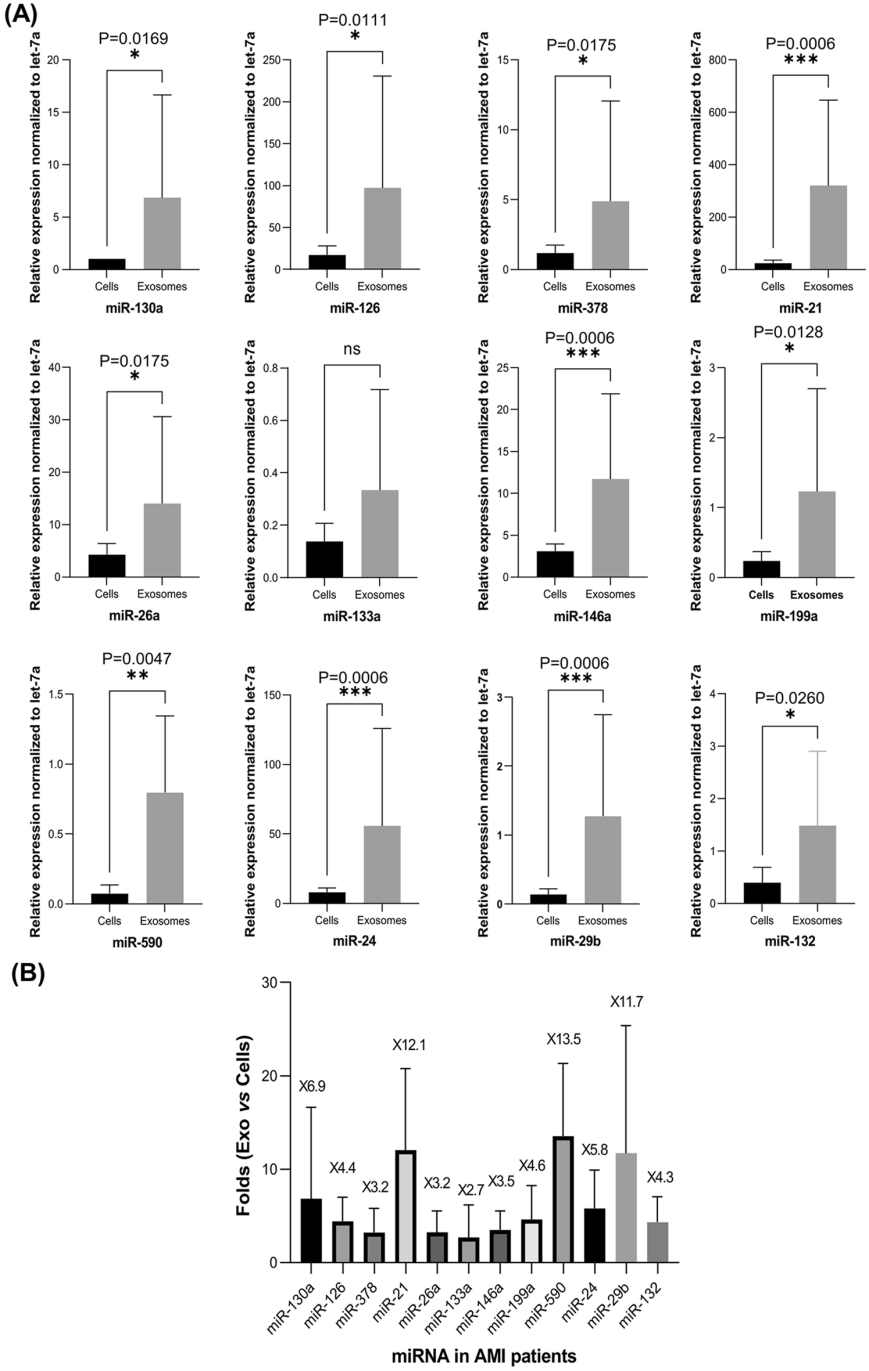
ProtheraCytes derived exosomes contain proangiogenic, anti-apoptotic, anti-fibrosis and cardiac miRNAs. (**A**) Total RNA from ProtheraCytes (Cells) and their exosomes (Exosomes) collected from AMI patients (n = 7) were subjected to real time PCR and normalized to small RNA (let-7a). miRNA expression in the exosomes secreted from ProtheraCytes was significantly higher than in ProtheraCytes (Mann–Whitney test). (**B**) Representation of the comparative fold change expression of exosomes versus cells for the indicated miRNAs (n = 7).

To investigate the proangiogenic activity of ProtheraCytes derived exosomes (CD34Exo) from AMI patients, we first isolated exosomes and characterized them by flow cytometry. We then isolated RNA from CD34Exo and determined the expression levels of miRNAs that have been reported to be key positive regulators of the angiogenesis processes. We assessed the expression levels of miR-126, miR-130a, miR-21, miR-378, and miR-26a^17,23–27^, in ProtheraCytes from AMI patients and their exosomes (CD34Exo). Exosomes were found to be significantly enriched in miR-130a, miR-126, miR-378, miR-26a, and miR-21, compared to ProtheraCytes (Fig. 3A). The miR-130a and miR-21 were the two most enriched miRNAs in CD34Exo and their expression was 6.9 and 12.1-fold higher, respectively, than in ProtheraCytes (Fig. 3B). Similarly, we observed significantly higher expression of miR-126 by 4.4-fold, and miR-378 and miR-26a by 3.2-fold in CD34Exo compared to ProtheraCytes from AMI patients (Fig. 3B). We also examined the expression of anti-angiogenic miRNAs (miR-34^28^, miR-143, and miR-506^29^) and observed that they were more enriched in CD34Exo but were expressed at much lower levels than the pro-angiogenic miRNAs (Supplementary Fig. 1).

These results indicate that ProtheraCytes are able to secrete exosomes containing proangiogenic miRNAs which might lead to the induction of angiogenesis and contribute to the vascular repair process after AMI. Interestingly, in addition to their involvement in angiogenesis, miR-21 and miR-26a have been also reported to inhibit myocardial cell apoptosis after AMI^30,31^. We also investigated the expression level of miR-146a, which has been shown to attenuate apoptosis and improve cardiac function following AMI^30,32^ and found that it was also enriched in CD34Exo by 3.5-fold compared to ProtheraCytes (Fig. 3B). Taken together, these data suggest that ProtheraCytes might protect cardiomyocytes from apoptosis immediately after AMI through the secretion of exosomes containing multiple anti-apopototic miRNAs.

We also investigated whether ProtheraCytes derived exosomes secrete miRNAs that promote myocardial regeneration, and evaluated the levels of miR-199a and miR-590, which have been described as playing an essential role in cardiac regeneration after myocardial infarction^33,34^. Expression of both miR-199a and miR-590 were detected at low levels but were significantly higher in CD34Exo compared to ProtheraCytes (Fig. 3A). Interestingly, miR590 was one of the most enriched miRNAs in CD34Exo with 13.5-fold higher expression and miR-199a was 4.6 times higher in CD34Exo than in ProtheraCytes (Fig. 3B).

Finally, we further examined the expression of miR-133a, miR-24, miR-29b and miR-132, known to have anti-fibrotic activity and been implicated in cardiac fibrosis^35–38^. As shown in Fig. 3A, B, miR-29b, miR-24, and miR-132 were significantly enriched in CD34Exo and their expression was 11.7, 5.8 and 4.3-fold higher, respectively, than in ProtheraCytes. On the other hand, there was no significant difference in the expression of miR-133 between CD34Exo and ProtheraCytes.

### ProtheraCytes express endothelial and cardiovascular markers

**Figure 4:**
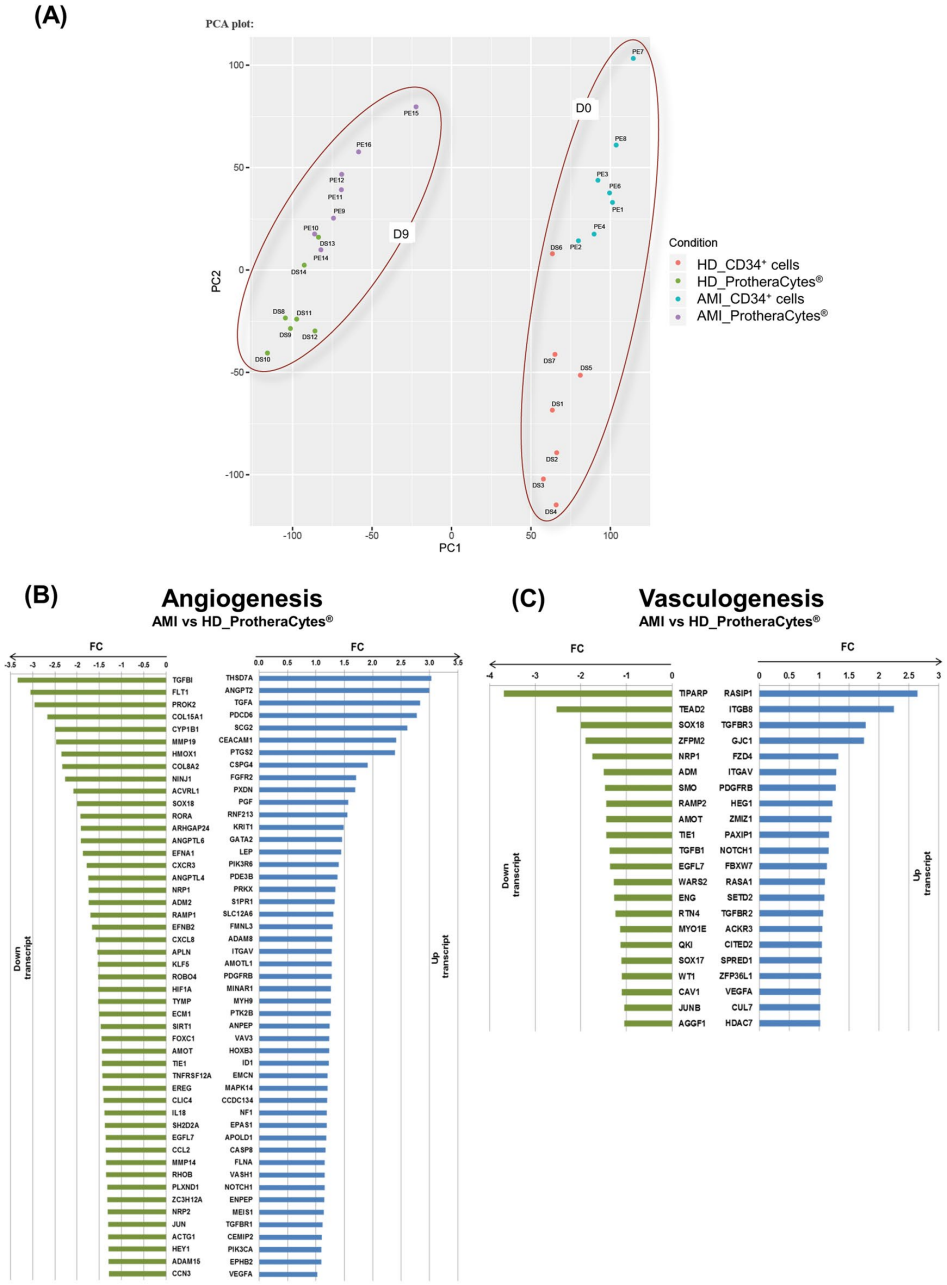
RNA expression levels detected by RNAseq analysis of ProtheraCytes from AMI patients versus healthy donors. PCA analysis (**A**)**:** the first dimension (PC1) separates the samples according to the time factor of cell expansion (D0, D9) and the second dimension (PC2) according to AMI or healthy donor (HD) group; Plot bar (FC) of up- and down- regulated genes involved in angiogenesis (**B**) and vasculogenesis (**C**) in D9 AMI patients vs healthy donor (HD) ProtheraCytes samples.

To further delineate the molecular signature of ProtheraCytes compared to naïve (unexpanded) CD34^+^ cells from AMI patients, a global analysis of mRNA expression was recently published by our group and revealed that ProtheraCytes express early markers of the cardiac and endothelial pathways and early cardiac-mesoderm markers^20^. We next sought to address whether ProtheraCytes also express angiogenic and vasculogenic markers. To test this, we compared the gene expression profiles of ProtheraCytes from AMI patients and healthy donors. As shown in the principal component analysis (Fig. 4A), we observed little variability between the AMI patient and healthy donor samples at day 9 but there was a clear separation between the samples at day 0 and day 9 of the cell expansion. Gene ontology analysis revealed the presence of two sets of transcripts grouping upregulated and downregulated genes involved in angiogenesis and vasculogenesis (Fig. 4B, C). However, most of them revealed little or no differential expression between AMI patients and healthy donors. Interestingly, some pro-angiogenic factors such as THSD7A (Thrombospondin type1 domain containing 7A), ANGPT2 (Angiopoietin-2), TGFA (transforming growth factor alpha), PDCD6 (Programmed Cell Death Protein 6) and SCG2 (Secreogranin II) were significantly upregulated by a factor of 3 in ProtheraCytes from AMI patients. Additionally, CEACAM1 (carcinoembryonic antigen related cell adhesion molecule-1), a key player in vascular development and angiogenesis and PTGS2, a gene involved in angiogenesis, (Prostaglandin Endoperoxide synthase 2) were also induced in AMI patients versus healthy donors. We also found some genes essential for blood vessel growth, e.g. RASIP1 (Ras-interacting protein1) and another involved in vasculature development (TGFBR3) with moderate enrichment in ProtheraCytes from AMI patients versus healthy donors (Fig. 4C). Furthermore, transcriptomic analysis of ProtheraCytes showed that there was no significant difference in VEGFA expression between AMI patients and healthy donors (Fig. 4B, C). This supports the previous finding showing that there was no significant difference in VEGF secretion between ProtheraCytes from AMI patients and healthy donors (Fig. 1B, C). Overall, these data suggest that expanded CD34^+^ cells express known angiogenic and vasculogenic genes that support their angiogenic properties and endothelial differentiation potential.

**Figure 5:**
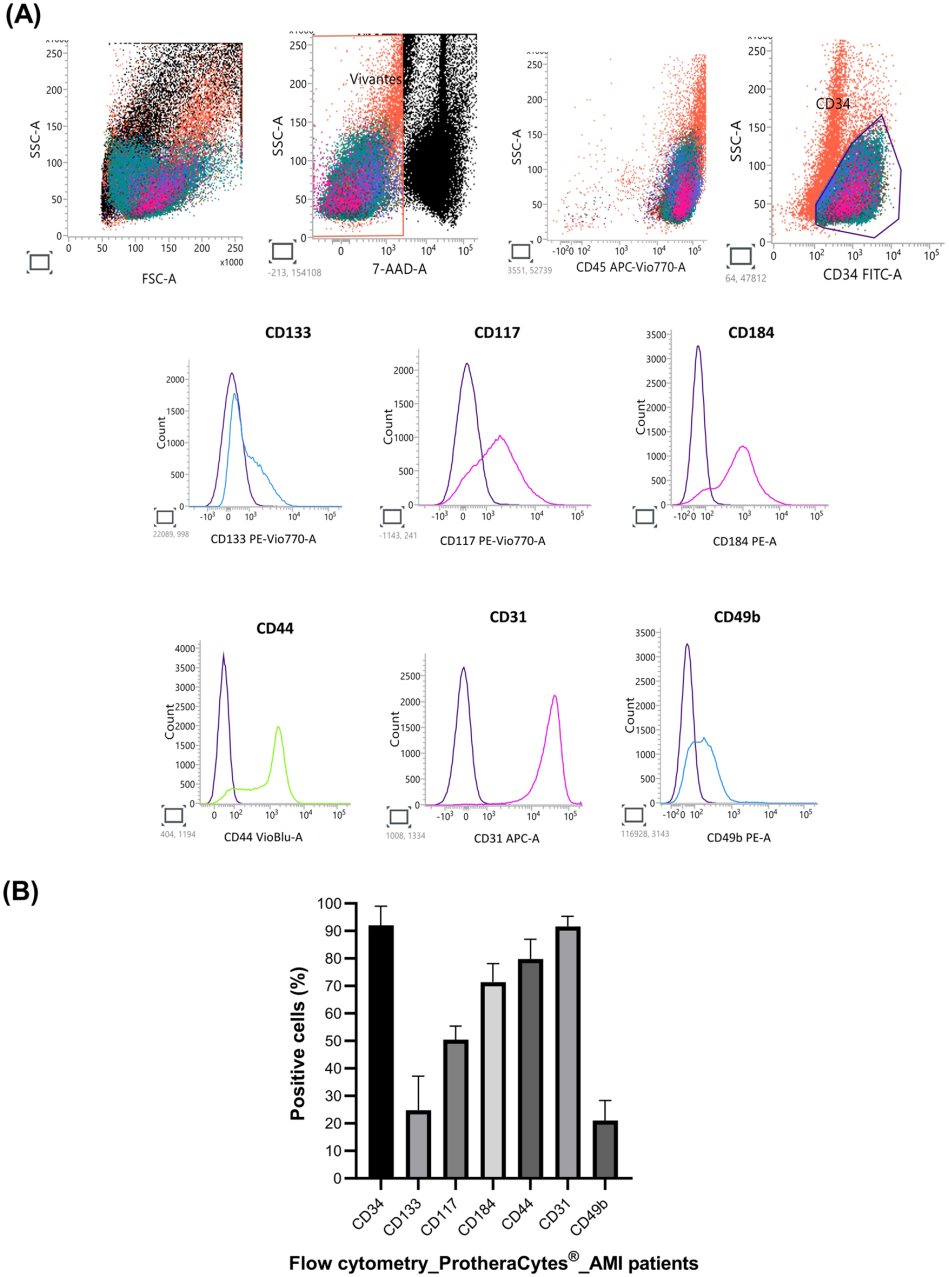
Flow cytometry characterization of ProtheraCytes cell surface markers. (**A**) Representative flow cytometry plots of ProtheraCytes from AMI patients. CD34^+^ cells were identified within mononuclear cells (MNCs) according to size criteria, reduction of background by stringent exclusion of dead cells, and lineage CD34^+^ cell phenotype by flow cytometry. (**B**) Histogram plots are shown with the corresponding isotype control antibodies (in purple) and the specific signal in cyan (CD133, CD49b), in magenta (CD117, CD184, CD31), and green for (CD144). Histogram represent the mean ± SD of (n = 10).

We next focused on analyzing the expression by flow cytometry of cell surface receptor markers previously found to be upregulated through our global study of mRNA expression by RNAseq^20^. According to the flow cytometry analysis, ProtheraCytes express stem cells markers such as CD133 (24.81%), CD117 (50.42%), CD184 (71.34%), CD44 (79.79%) and endothelial markers CD31 (91.63%) and CD49b (21.08%) (Fig. 5). It is important to note that CD133, CD44, and CD49b markers and can be found in other cell types.

### ProtheraCytes display endothelial differentiation potential in vitro

**Figure 6:**
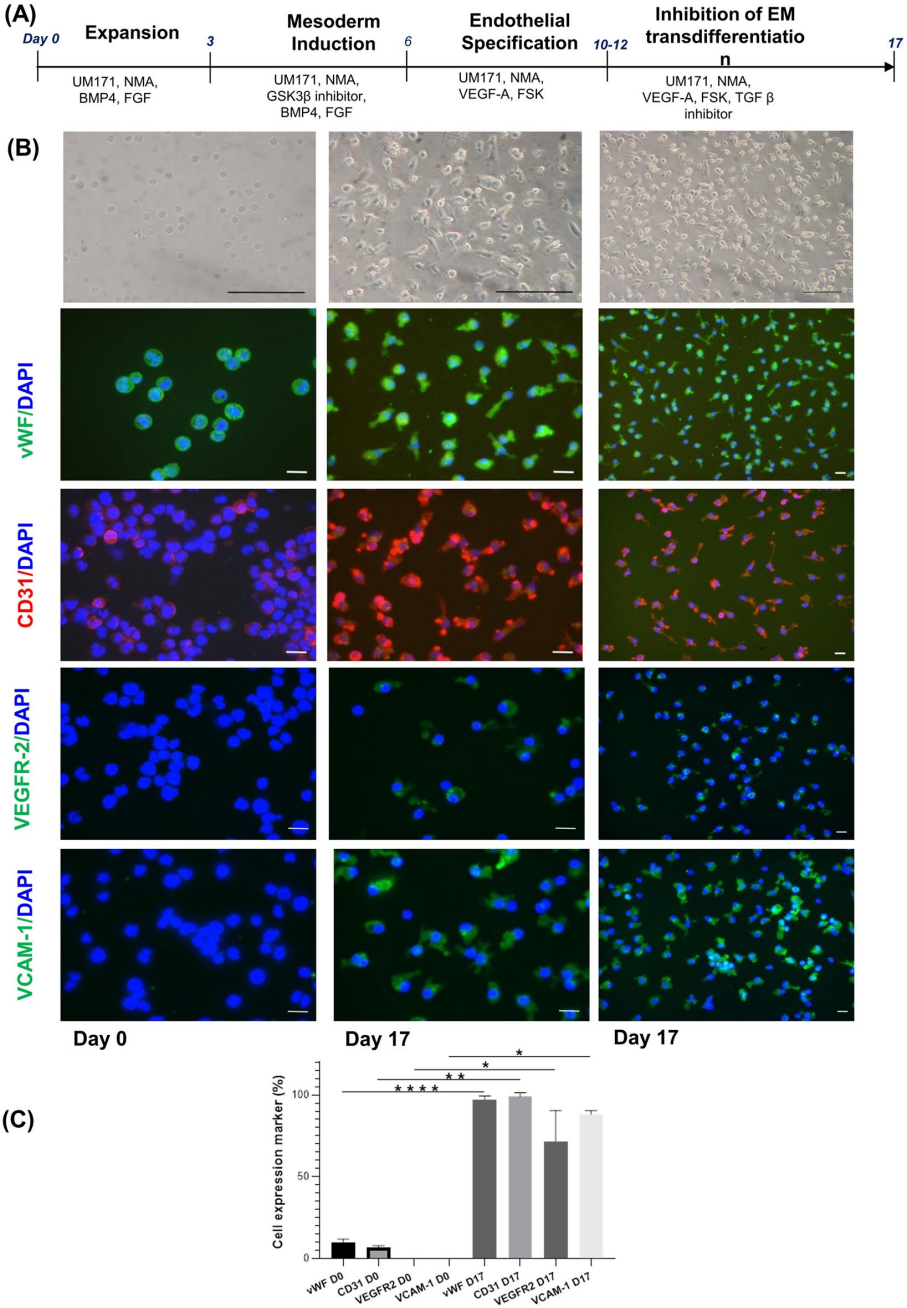
Differentiation of ProtheraCytes into endothelial cells. (**A**) Schematic representation of the differentiation protocol of ProtheraCytes into endothelial cells. (**B**) Upper panel—bright-field image of ProtheraCytes at day 0 and endothelial differentiation at day 17. Scale bar, 100 µm. Bottom panel—immunofluorescence staining showing positive staining for either endothelial marker vWF, CD31 (PECAM1), VEGFR-2, or VCAM-1 of ProtheraCytes and differentiated ProtheraCytes at day 0 and day 17. Green is Alexa Fluor 488 stained vWF, VEGFR-2 or VCAM-1 and red is Alexa Fluor 555 stained CD31. DAPI was used to visualize nuclei. The pictures display representative merged immunofluorescence images. Scale bar, 20 µm. (**C**) Histogram plots showing percentage of positive cells at day 0 and day 17 (*p < 0.05, **p < 0.01, ****p < 0.0001).

We have shown thus far that ProtheraCytes secrete paracrine factors involved in angiogenesis, survival and cardiac regeneration that may promote endothelial and cardiac repair of damaged myocardial tissue. We next thought to address whether ProtheraCytes have the potential to differentiate into endothelial cells. To test this hypothesis, ProtheraCytes were subjected to endothelial differentiation using a multistep differentiation strategy and compounds inducing expansion, reprogramming and differentiation^22^ (Fig. 6A). Distinct morphological changes were observed while the cells differentiated into the endothelial lineage (Fig. 6B). After 10 days of differentiation, cells adhered to the substrate and showed hallmarks of endothelial cells, including extended elongated morphology. At 17 days of culture, greater numbers of cells displayed the typical morphology of endothelial cells (Fig. 6B). To characterize them, we performed immunofluorescence staining in the cells primed to commit to the endothelial lineage and in the undifferentiated ProtheraCytes to monitor the expression of endothelial markers CD31, von Wilbrand factor (vWF), VEGFR-2 and VCAM-1. Immunostaining showed that at day 17, the differentiated cells co-expressed vWF (97.2 ± 2.3%), CD31 (98.9 ± 2.5%), VEGFR-2 (71.5 ± 19.1%) and VCAM-1 (88.2 ± 2.4%) (Fig. 6C). Importantly, the expression of CD31, vWF, VEGFR2, and VCAM-1 was significantly increased compared to ProtheraCytes before differentiation. Collectively these data demonstrate the differentiation potential of ProtheraCytes into endothelial cells in vitro.

## Discussion

As a global industrial standard for cell therapy products (CTPs), the evaluation of potency plays a key role in defining the quality of the CTP. The development of the potency assay is based on the quantitative measure of a relevant biologic function linked to the mechanism of action of the CTP for that particular disease indication. The potency assay is a critical quality control measure required for batch release to demonstrate its biological activity and guarantee quality, consistency, and stability between batches^39^. To select and develop a consistent and reliable potency assay, we characterized the ProtheraCytes gene expression, growth factor secretion, in vitro functionality, and miRNA expression in secreted exosomes.

First, we showed that ProtheraCytes secrete paracrine factors such as VEGF that can promote endothelial and cardiac repair of damaged myocardial tissue. Indeed, the concentration of VEGF found in the culture supernatants was significantly correlated to the numbers of CD34^+^ cells harvested after the manufacturing of ProtheraCytes. We also showed that the ProtheraCytes supernatant containing VEGF has angiogenic activity as shown by the in vitro tube formation assay with HUVECs. We performed the same experiment with VEGF alone and observed comparable results to the ProtheraCytes supernatant. In vivo, the angiogenic properties of VEGF have been demonstrated in rodent^40^ and porcine models of MI^41,42^. In rodents, hearts injected with DNA encoding VEGF showed increased focal epicardial blood vessel density^40^ and in pigs, VEGF infusion resulted in better ejection fraction and regional wall thickening^41^. In an experimental nude-mouse model of MI, Wang and colleagues showed that intramyocardial injection of human CD34^+^ cells in the peri-infarct region significantly increased the left ventricular ejection fraction and the formation of new blood vessels^43^. They showed that this recovery was mediated by the CD34^+^ cell secretion of VEGF, because when they added anti-VEGF antibodies, which abolished the formation of human-derived endothelial cells and the paracrine effect mediated by VEGF, it abrogated the improvement in left ventricular ejection fraction. Concluding that angiogenesis and paracrine effect are responsible for the improvement in cardiac function observed after CD34^+^ cell therapy. Clinical studies have also shown that chronic myocardial ischemia patients transfected with a plasmid encoding VEGF experienced reduced angina and ischemia and improved myocardial perfusion^44^.

Secondly, we characterized ProtheraCytes-derived exosomes and determined that their average size is 86.7 ± 10.17 nm, in line with previously reported results for CD34^+^ cell-derived exosomes^17^. These ProtheraCytes-derived exosomes express the characteristic exosomal markers CD63, CD81 and CD9 as well as endothelial and stem cell markers. Exosomes contain a variety of molecules, including proteins, lipids, DNA, mRNA, long noncoding RNA, circular RNA and miRNAs. Among them, exosomal miRNAs are the most numerous exosomal cargo molecules^45^. The underlying mechanism of how miRNAs are taken up by exosomes is not entirely clear, but four possible pathways for miRNA sorting into exosomes have been identified: the sphingomyelinase 2-dependent pathway, heterogeneous nuclear ribonucleoprotein-dependent pathway, the miRISC-associated pathway, and the sequence-dependent pathway involving the 3′-end of miRNA^45–47^. Even though the size of the exosomes is larger than the intercellular space of the endothelium, most of their cargo is released through direct fusion with the target cell’s membrane, endocytosis, receptor-ligand interaction and phago/ pinocytosis^48,49^. We then examined whether exosomes from ProtheraCytes contain proangiogenic and antiapoptotic miRNA that may promote myocardial regeneration. We isolated and measured the miRNA levels from exosomes collected from ProtheraCytes and observed that CD34Exo are particularly enriched in miRNAs involved in vascular development, such as miR-21 and miR-146, which may exert synergistic angiogenic effects^30^. Exosomes enriched with miR-21 have been shown to have cytoprotective activity in vitro in neonatal cardiomyocytes and support enhanced microvessel density in vivo in a rat model of MI^50^.

miR-126 and miR-130a were also enriched in CD34Exo. Luo and colleagues have shown that miR-126-enriched exosomes decrease myocardial cell injury and expression of fibrosis-related proteins under hypoxic conditions and promote microvascular generation and migration in vitro^51^. In AMI rats, miR-126-enriched exosomes significantly decreased the myocardial injury area of infarction, cardiac fibrosis, and inflammatory cytokine expression and promoted blood vessel formation^51^. miR-130a has been shown to promote angiogenesis by downregulating the expression of homeobox A5 (HOXA5) and growth arrest-specific homeobox (GAX)^52^. We found that CD34Exo contain anti-angiogenic miRNAs^53^ (miR-34a, miR-143 and miR-506) but their expression level is much lower than the pro-angiogenic miRNAs.

We also identified other miRNAs enriched in the Protheracytes derived exosomes (miR-199a, miR-590) that have the ability to induce therapeutic regeneration of damaged tissue and were reported to play an essential role in heart development, disease and regeneration^18,54,55^. Furthermore, we identified antifibrotic miRNAs (miR-133a, miR-24, miR-29b and miR-132) in CD34Exo with cardiac antifibrosis activity^35–38^. Lastly, other miRNAs such miR-210, miR-23a-3p, miR-30b, miR-30c and miR-424 have been shown to improve heart function after myocardial infarction by promoting cardiac angiogenesis and vasculogenesis^56^.

We then analyzed the expression profile of angiogenic and vasculogenic genes in ProtheraCytes from AMI patients and healthy volunteers and found only small differences between them. Only a few proangiogenic genes such as THSD7A, ANGPT2, and TGFA and vasculogenic genes including RASIP1 and TGFBR3 were upregulated in AMI patient samples. Flow cytometry characterization showed that ProtheraCytes express stem cell and endothelial surface markers, although some of these markers are also expressed by other cell types. Finally, as CD34 is a marker of endothelial progenitor cells^57^ with the potential to regenerate ischemic tissues^58–61^, we sought to address the in vitro differentiation potential of ProtheraCytes into endothelial cells following a multistep differentiation strategy^22,62^. Immunofluorescence analysis of the cell cultures on day 17 of differentiation showed that differentiated cells expressed higher levels of the endothelial markers CD31, vWF, VEGFR-2, and VCAM-1 compared to control ProtheraCytes and underwent clear morphological changes characteristic of endothelial progenitor cells.

Our data showed that ProtheraCytes are able to secrete paracrine factors, such as VEGF and miRNAs that may play a role in angiogenesis, survival and cardiac regeneration, promote endothelial repair, and decrease apoptosis of damaged myocardial tissue. Based on the evaluation of all the data, we chose the VEGF ELISA as a potency assay, because it is a fast, reliable, quantitative assay that allows for the timely batch release before ProtheraCytes are shipped to the clinic for injection. This potency assay has been previously used by other sponsors in cell therapy trials for acute myocardial infarction^63^ and critical limb ischemia^64^, and follows the International Society for Cell & Gene Therapy guidelines for potency assay development^39^.

### Supplementary Information

Supplementary Figure S1.

## Data Availability

All data associated with this study are available in the main text or uploaded to GEO (accession# GSE220865).
