# Supplementary material for: Development of a potency assay for CD34+ cell-based therapy
Source: Sci Rep. 2023 Nov 11;13:19665. doi: 10.1038/s41598-023-47079-8 (PMC10640600; doi:10.1038/s41598-023-47079-8)
Supplement: Supplementary file 1 — Supplementary Figure S1. [file 41598_2023_47079_MOESM1_ESM.pptx]

## Slide 1
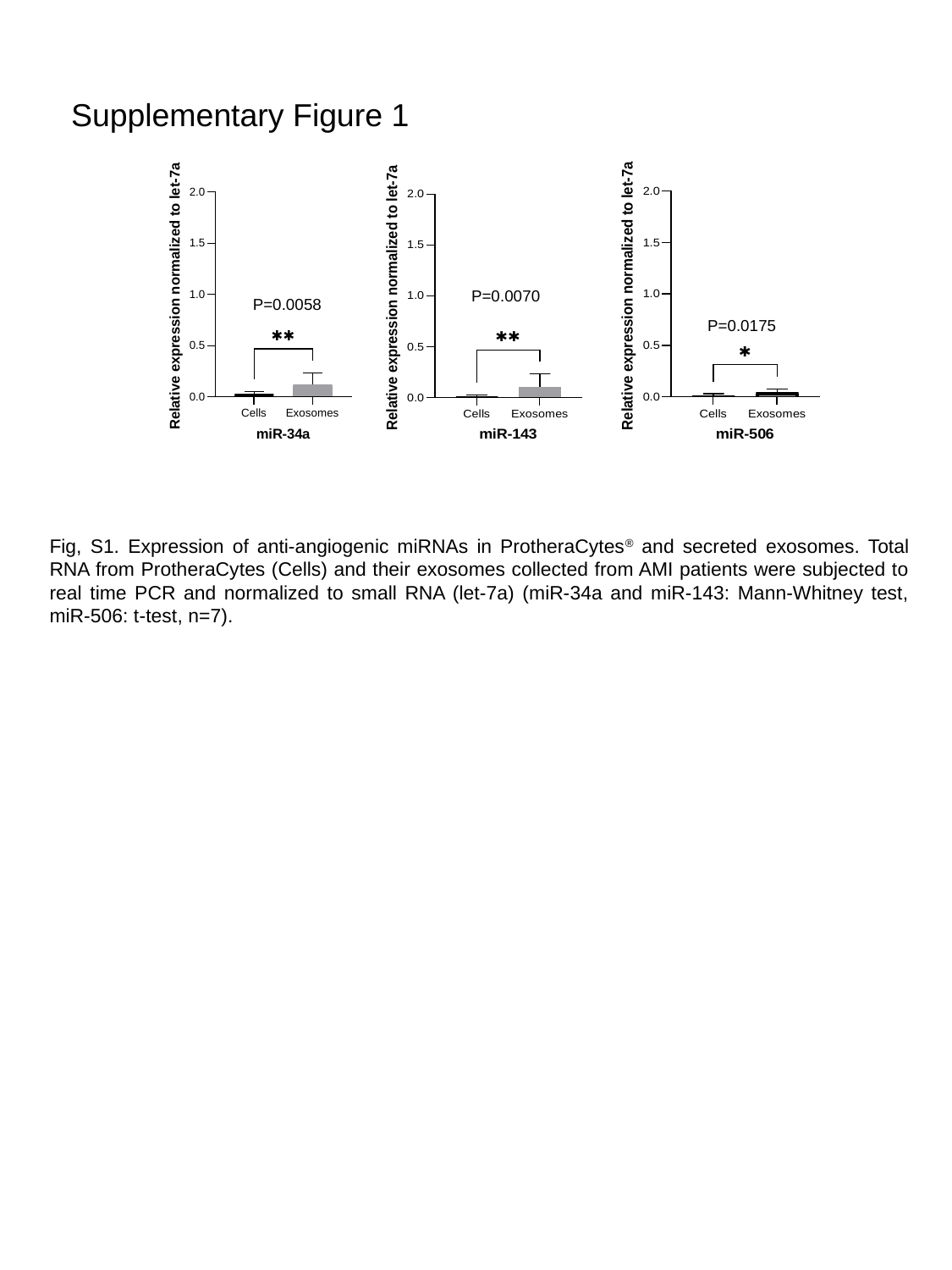

Supplementary Figure 1
P=0.0070
P=0.0058
P=0.0175
Fig, S1. Expression of anti-angiogenic miRNAs in ProtheraCytes® and secreted exosomes. Total RNA from ProtheraCytes (Cells) and their exosomes collected from AMI patients were subjected to real time PCR and normalized to small RNA (let-7a) (miR-34a and miR-143: Mann-Whitney test, miR-506: t-test, n=7).
